# The circadian clock has roles in mesenchymal stem cell fate decision

**DOI:** 10.1186/s13287-022-02878-0

**Published:** 2022-05-16

**Authors:** Wenzhen Gao, Rong Li, Meilin Ye, Lanxin Zhang, Jiawen Zheng, Yuqing Yang, Xiaoyu Wei, Qing Zhao

**Affiliations:** 1 State Key Laboratory of Oral Diseases & National Clinical Research Center for Oral Diseases, West China Hospital of Stomatology, Sichuan University, Chengdu, 610041 China; 2 Shandong Key Laboratory of Oral Tissue Regeneration & Shandong Engineering Laboratory for Dental Materials and Oral Tissue Regeneration, School and Hospital of Stomatology, Shandong University, Jinan, 250012 China

**Keywords:** Circadian clock, Mesenchymal stem cells, Cell fate decision, Transcription factors, Epigenetics

## Abstract

The circadian clock refers to the intrinsic biological rhythms of physiological functions and behaviours. It synergises with the solar cycle and has profound effects on normal metabolism and organismal fitness. Recent studies have suggested that the circadian clock exerts great influence on the differentiation of stem cells. Here, we focus on the close relationship between the circadian clock and mesenchymal stem cell fate decisions in the skeletal system. The underlying mechanisms include hormone signals and the activation and repression of different transcription factors under circadian regulation. Additionally, the clock interacts with epigenetic modifiers and non-coding RNAs and is even involved in chromatin remodelling. Although the specificity and safety of circadian therapy need to be further studied, the circadian regulation of stem cells can be regarded as a promising candidate for health improvement and disease prevention.

**Supplementary Information:**

The online version contains supplementary material available at 10.1186/s13287-022-02878-0.

## Background

**Fig. 1:**
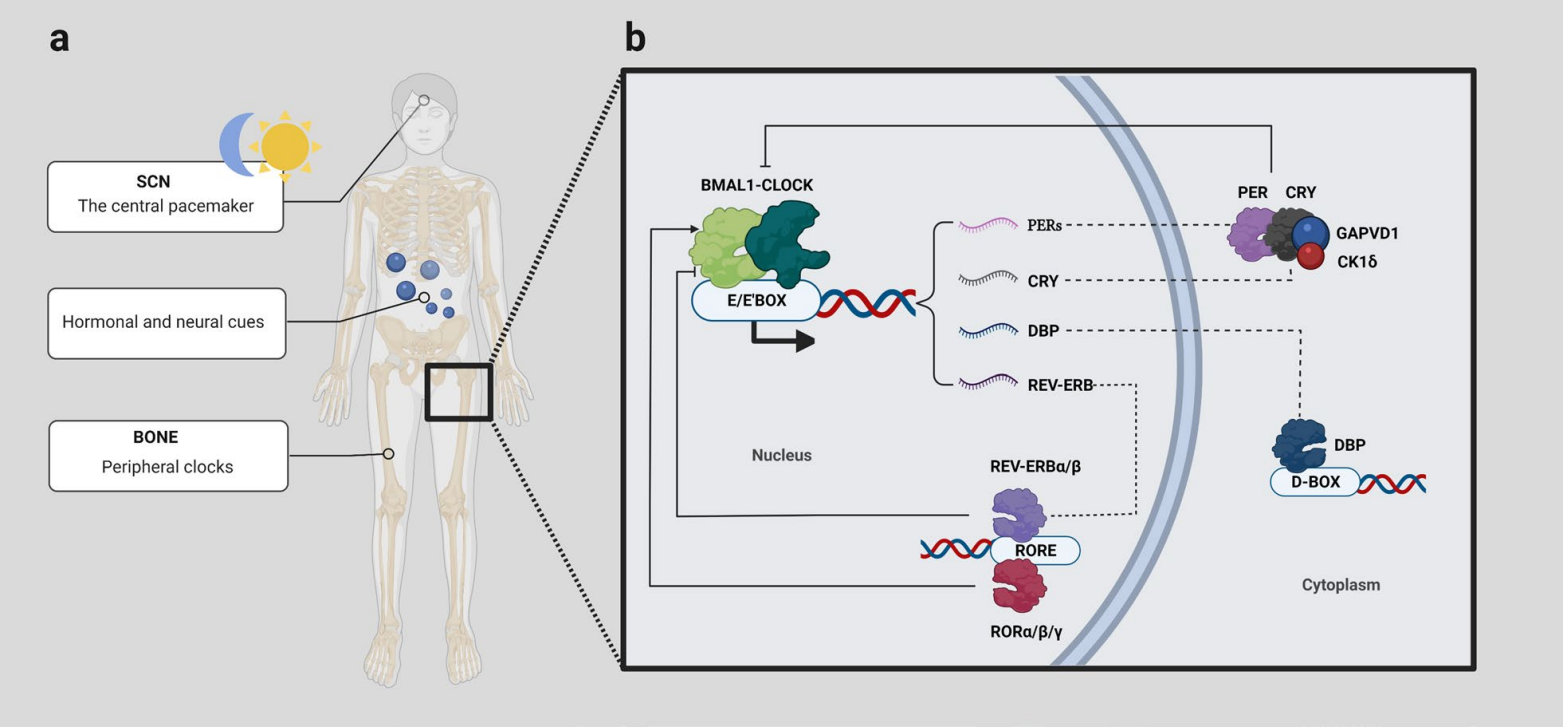
**a** The circadian clock is distributed throughout the body. The retina perceives light information, which will be relayed to the SCN. In turn, the SCN entrains peripheral system clocks via neural, humoral, and metabolic cues [6]. **b** The multilevel transcription–translation feedback loop of circadian rhythm. The central loop is based on reciprocal regulation between the BMAL1-CLOCK complex and the PER complex. BMAL1 and CLOCK bind to E-boxes in the promoters of a subset of clock-controlled genes (CCGs) in a heterodimer form, promoting the expression of *Per*, *Cry*, *Rev-erb*, etc. Once PERs and CRY in the cytoplasm accumulate to a certain extent, they will enter the nucleus under GAPVD1 modulation and suppress the transcriptional activity of the BMAL1-CLOCK complex [7]. Casein kinases 1δ/ε phosphorylate PERs, which affects their nuclear translocation and stability [8]. In the second transcription loop, nuclear receptors RORα/β/γ and REV-ERBα/β compete for the ROR element of BMAL1 [9]

Circadian rhythm, also known as the circadian clock, directs and maintains nearly 24-h periodic changes in behaviour and physiology [1]. The central pacemaker of endogenous circadian clocks lies in the SCN (suprachiasmatic nuclei) of the anterior hypothalamus, which is sensitive to environmental cues (called zeitgebers) and orchestrates peripheral synchronicity through hormonal and neural mechanisms [2, 3]. Light input is one of the essential entraining daily oscillations and triggers the sympathetic release of norepinephrine that synchronises the peripheral clocks (Fig. 1a) [4]. The molecular clock is essentially a multilevel transcription–translation feedback loop that integrates a number of regulatory cues, including *Bmal1* (brain and muscle ARNT-like protein 1/aryl hydrocarbon receptor nuclear translocator-like protein 1), *Clock, Per* (period), *Cry* (cryptochrome), *RORα/β/γ* (retinoid-related orphan receptor α/β/γ), and *Rev–erbα/β* (Fig. 1b) [5].

The circadian clock has a great influence on the differentiation process in stem cells derived from different sources [10]. *Bmal1 knockdown* changes the stem cells’ differentiation potential with an aberrant induction of differential gene expression in vitro, and it is essential for transcriptional programmes in cell differentiation [11]. However, how mesenchymal stem cell (MSC) differentiation responds to rhythmic changes timely is a fundamental question to be answered in bone metabolism. Vast amounts of research continue to emerge and are yet to be summarised. This review links new developments to earlier findings, aiming to clarify how circadian clock genes regulate stem cell differentiation fate in the skeletal system and hoping to exploit the MSC differentiation potential by targeting the circadian clock. This review opens up the possibility of a new strategy for MSC regulation and will potentially improve MSC differentiation efficiency for future clinical applications.

1. **Stem cells and the circadian clock**

Prior to the late 1970s, researchers sought to interrogate how stem cells controlled by the circadian clock perform tissue homeostasis and regenerative functions. Today, rhythmicity has been shown to be instrumental in stem cells’ self-renewal and activation in the event of injury and mammalian development [12]. Multiple studies have confirmed that circadian components exist in undifferentiated ESCs (embryonic stem cells), even before zygotic genome activation [13, 14]. However, ESCs show non-oscillating clock gene expression and are thought to lack clock function. A self-sustained oscillator occurs during postnatal development with the gradual appearance of CLOCK, which is controlled by Dicer/Dgcr8-mediated post-transcriptional mechanisms [15, 16]. Interestingly, Paulose et al. [17] identified the rhythmicity of glucose utilisation and glucose transporter mRNA in ESCs in the absence of clock gene oscillation. This meant that circadian variations in some physiological activities are independent of the core clock, such as peroxiredoxin hyperoxidation [18]. Additionally, there may be other clock regulatory mechanisms in ESCs, and core clock genes may have a non-clock function in ESCs. The deletion of *Clock* reduces proliferation and increases apoptosis in ESCs, while *Bmal1* has been demonstrated to be essential for a proper lineage differentiation of ESCs. Depletion of *Bmal1* adversely affects the subsequent differentiation, leading to an imbalance of lineage-specific or selective activation of genes with increased pluripotency transcription factor Nanog, which may be closely related to the change of PI3K, MAPK, and Wnt pathways [19].

Although induced pluripotent stem cells (iPSCs) do not display oscillating clock genes, expression rhythms arise spontaneously in their directed differentiation process [16], which can also be reversed if iPSCs are reprogrammed to pluripotency. This indicates that clock function may be a common feature of differentiated and mature cells [20].

Multipotent stem cells from different tissues show differential clock gene expression profiles with different amplitude ranges. For example, compared with bone marrow stem cells (BMSCs) and adipose tissue-derived mesenchymal stem cells (ADSCs), dental pulp stem cells (DPSCs) have higher *Bmal1* expression. After circadian synchronisation by dexamethasone, PER2 peaked at 32 h in BMSCs, with a nadir at 16 h in ADSCs [10]. The circadian clock and daily changes in light and darkness operating at the RNA and protein levels control the proliferation and differentiation of various multipotent cells such as cardiac progenitor cells, stem cells from the epidermis, hair follicle, nervous system, and haematopoietic tissue [4, 12]. For example, the rhythmic *Per and Cycle* underlie fate decisions and regeneration of intestinal epithelial stem cells [21].

2. **MSC differentiation exhibits a circadian pattern in the skeletal system**

The circadian regulation of the skeletal system and the differentiation of MSCs have been widely recognised [22]. Luciferase reporter assays detect the 24-h rhythm of OCN mRNA, an osteogenesis marker [23]. BMP2 and RUNX2 change with the light cycle and the level of melatonin [24]. Serum bone turnover markers also show diurnal fluctuations [25], and the irregular light–dark cycle experienced by pregnant mice will affect the offspring’s circadian rhythm and lead to decreased bone perimeter, endocortical volume, and bone strength [26]. The dynamic balance of bone formation and resorption under strict time control ensures bone mass integrity and bone structure and function [27]. The fate change in MSCs resulting from a disrupted circadian clock is implicated in osteoporosis, elevated fracture risk, and osteoarthritis. Continuous exposure to artificial light significantly increases osteoporosis risk in humans and animals [28], and shift workers have a higher fracture risk [29]. Bioinformatic analysis of osteoarthritis patients’ transcriptome shows that clock imbalance may be the leading cause for these complications [30].

The intrinsic molecular clock underlies MSC differentiation at the genetic level. Clock gene (*Bmal1*, *Pers*, etc.) knockout and knockdown lead to the phase change of MSC differentiation. Disruption of *Clock* and *Per2* results in a significant reduction in adipogenic differentiation without apparent changes in osteogenic differentiation [31]. BMAL1 oscillates in skeletal tissue and seems to promote osteogenesis and inhibit adipogenesis in a model-dependent manner. *Bmal1−/−* mice show low bone mass phenotypes with decreased bone trabeculae, bone density, and a thinner cortical bone, resulting from osteogenic MSC differentiation defects [32, 33], and the BMP2 signal may be a key target for BMAL1 [34]. *Per−/−* *and Cry−/−* mice show a significant increase in bone mass in both vertebrae and long bones [35]. This high bone phenotype worsens over time [36]*.* REV-ERBα, a negative regulator of BMAL1, gradually decreases with MSC osteogenesis, and its overexpression reverses the process [37]. However, the results are contradictory. ROR*α,* a positive regulator of BMAL1, increases with MSC osteogenesis and late adipogenesis [38]. *Bmal1* interference promotes adipogenesis with Wnt pathway down-regulation in preadipocytes. A high expression pattern of Bmal1 has been testified in mature adipocytes [39]. The knockdown of Bmal1 by RNA interference in mature 3T3-L1 cells only leads to a small amount of lipid droplet formation [40]. These findings suggest the distinct roles of the circadian clock at different differentiation stages that require further research.

### The circadian clock regulates MSC differentiation by targeting hormones

Melatonin, synthesised in the pineal gland, is mainly stimulated by darkness and inhibited by light [41]. It keeps the bodily rhythms synchronised with the outside environment and has a positive effect on bone health [42, 43]. Decreased melatonin levels caused by ageing will increase the probability of adverse skeletal events [44], and this can be rescued by its timed administration. Melatonin’s capacity to reduce MSC oxidative stress-induced toxicity and stress-induced cellular senescence by increasing SIRT1 (sirtuin1) [45, 46] provides further evidence on its positive role in promoting bone health [47, 48]. It drives MSCs into an osteoblast lineage by inhibiting miRNA sponge circ-0003865 [49] and by taking part in osteogenesis signalling cascades, such as p38, BMP2, JNK, and Wnt-βcatenin pathways [50, 51]. Melatonin also increases chondrocyte cell volume and enhances chondrogenic differentiation through the Wnt pathway [52].

Parathyroid hormone (PTH) resets the skeletal clock by up-regulating *Per* mRNA expression [53], and its concentration in bodily fluids exhibits a circadian rhythm. PTH has been widely used in cartilage tissue engineering in recent years, as it inhibits chondrogenic differentiation but mitigates cartilage degeneration. Intermittent PTH protects cartilage by attenuating chondrocyte hypertrophy in 3D-printed tissue engineering scaffolds [54], although it reduces MSC cartilage terminal differentiation markers [55]. The deletion of PTH receptors in MSCs leads to decreased bone formation and increased bone marrow adipose tissue (BMAT), and intermittent PTH administration significantly reduced BMAT in control mice, also found in male patients with osteoporosis [56]. Research identifies that the number of bone marrow adipocytes in men with idiopathic osteoporosis decreased by 27% after 18 months of PTH treatment [56].

Glucocorticoids (GCs) mediate the transcription of *Per1* and reset of a peripheral circadian rhythm [57]. SCN controls GC levels in plasma via the hypothalamic–pituitary–adrenal axis, and *Per2* may be critical for this process [58]. Oscillation amplitudes of GCs are a crucial determinant of their biological function. The periodic GC-activated GC receptor directly influences the differentiation and functional integration of stem cells in hippocampi. GCs are directly or indirectly tied to osteoporosis by regulating PER2 [59]. Besides, GCs change the methylation profile of cell cycle regulation and Wnt signalling pathway genes [60].

### The molecular clock influences MSC multilineage differentiation by regulating transcription factors

Various transcription factors spatiotemporally control the MSC differentiation in response to different stimuli. They directly interpret the genome, and their binding to it is the first step in DNA decoding. In bone metabolism, osteogenic factors, like RUNX2, guide MSCs to the osteogenic lineage; PPARγ promotes adipogenic differentiation; SOX9 participates in chondrogenesis. These transcription factors form a transcription network that plays a crucial role in MSC fate decisions. Network imbalance may lead to skeletal malformations and bone mass disorders. On the other hand, the mechanisms underlying these factors may provide solutions for the treatment of bone diseases.

#### Regulation and role of transcription factors in osteogenesis

RUNX2 is the ‘master switch’ of osteogenic differentiation, with a highly conserved DNA-binding runt domain. *Runx* −/− mice show a lack of osteogenic markers and complete blockage of intramembranous and endochondral ossification [61]. Cleidocranial dysplasia resulting from the mutation of RUNX2 is characterised by skeletal dysplasia, such as clavicle hypoplasia and abnormal clavicle [62]. Surprisingly, the overexpression of RUNX2 also shows bone defects in mice, including a dramatic reduction in osteoblast numbers with immature phenotypes. Therefore, RUNX2 plays a major role in MSCs’ early lineage commitment to osteoblasts and maintains the latter at an earlier stage to provide a large number of immature osteoblasts.

RUNX2 shows expression rhythmicity in various tissues, including the SCN and bone [63], and is involved in connecting the circadian clock and MSC fate decision. RUNX2 influences enamel formation in MSCs under the control of circadian pacemaker [64]. Some researchers suppose that RUNX2 relies on the activity of BMAL1, as the rhythm of RUNX2 mRNA disappears in mouse SCN without *Bmal1* [63]. Still, some believe REV-ERBα is the upstream regulator of RUNX2 and disturbs the recruitment of RUNX2 to the targeted promoter. Knockdown of REV-ERBα enhances the differentiation and function of pre-osteoblasts [65].

**Fig. 2:**
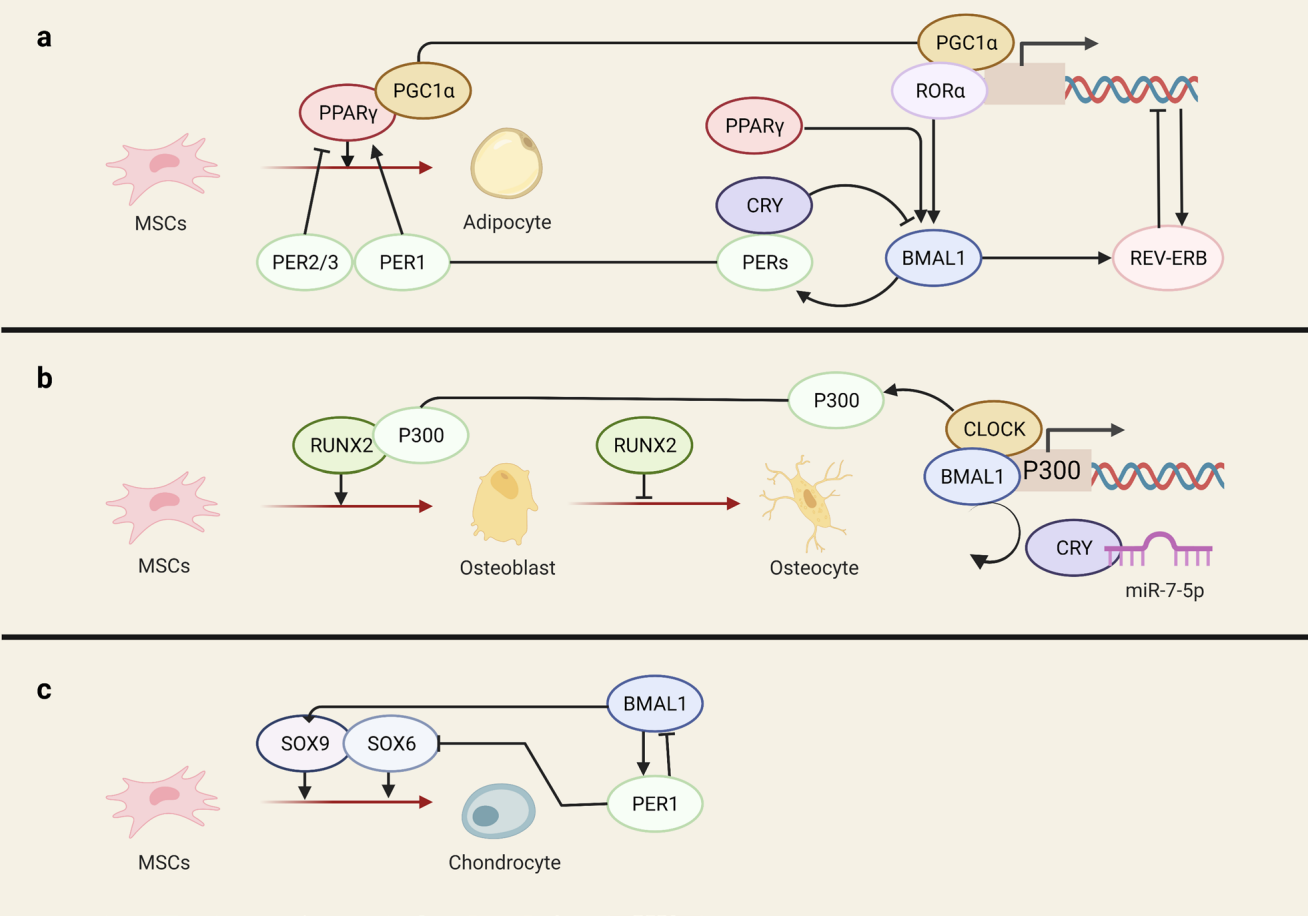
**a** Both PPARγ and its cofactor PGC-1α serve as master transcription factors in adipogenic differentiation and are tightly regulated by the circadian clock. **b** miR-7-5p liberates CRY from the BMAL1-CLOCK complex to promote the transcription of P300. P300 and RUNX2 act simultaneously on the targeted promoter. **c** BMAL1 promotes the expression of SOX9 and PER1 inhibits SOX6 expression

miRNA is also one of the primary mechanisms underlying the rhythm of RUNX2. miR-7-5p disentangles CRY2’s inhibition on the BMAL1-CLOCK complex. The released BMAL1-CLOCK stimulates P300 transcription, promoting histone H3 acetylation and forming a transcription complex with RUNX2 to enhance bone formation (Fig. 2) [66]. Another miRNA, miR-433, shows robust circadian rhythmicity with a peak after dark and targets RUNX2 3’-UTR causing its degradation [67]. In miR-433-inhibited mice skulls, the mRNA levels of RUNX2 and its downstream osteocalcin increased significantly [68].

Besides acting as a transcription factor, RUNX2 also indirectly regulates the transcription of clock genes and/or clock-controlled genes (CCGs) by working as a scaffold to recruit growth factors and cytokines [63, 69], including P300, CCAAT/enhancer-binding protein, and histone deacetylase [70], with its protein–protein interacting domains.

To our knowledge, Osterix, cAMP-dependent transcription factor 4 (ATF4), Forkhead box O (FOXO), and other transcription factors in osteogenic differentiation have rarely been described in circadian rhythm studies. The role of these transcription factors in circadian rhythm remains to be identified.

#### Adipogenic master regulator PPARγ

Peroxisome proliferator-activated receptor γ (PPARγ) belongs to the nuclear hormone receptor superfamily with the highest expression in adipose tissue. It plays a vital role in the balance between osteogenic and adipogenic differentiation of MSCs [71–73]. PPARγ up-regulation promotes adipogenic differentiation and inhibits the generation of osteoblasts and chondrocytes [74], with its down-regulation having opposite effects [75]. Besides, the PPARγ agonist thiazolidinedione (TZD) is associated with bone loss and fracture [76]. Clinical experience and animal studies suggest that the fate change of MSCs associated with PPARγ may be the main reason for this.

PPARγ and its cofactor PGC-1α display a clear circadian expression pattern in many tissues, and PPARγ even acts as a crucial peripheral clock activator of the cardiovascular system and metabolism [77, 78]. Multiple randomised clinical trials have shown that the clock hormone melatonin up-regulates PPARγ [79, 80]. And the inhibition of *Clock* or *Per2* changes the expression of osteocalcin in MSCs, in association with extraordinary increases in PPARγ [31]. BMAL1 and REV-ERBα are induced by PPARγ and PGC-1α [77, 81, 82]. PPARγ is deeply involved in the regulatory networks between circadian rhythms and MSC fate decisions (Fig. 2), with the mechanisms described hereafter.

Nocturnin*−/−* mice show deregulated circadian patterns in PPARγ gene expression and increased bone mass [83]. Nocturnin adapts to daily environmental changes by changing the organism’s metabolic state [84, 85]. It works as a pivotal mediator of mitochondrial function by dephosphorylating nicotinamide adenine dinucleotide phosphate (NADP+/NADPH) [86]. Nocturnin is highly expressed in MSCs, and its overexpression enhances the lipogenesis of preadipocytes 3T3-L1. It enhances PPARγ transcription activities and guides PPARγ nuclear translocation.

*Per1* and *Per2* have antagonistic influences on PPARγ activity. PER1 enhances PPARγ activity operating as its natural modulator, while PER2 directly and specifically inhibits the recruitment of PPARγ to the target promoter [87]. However, the PER2 regulation of adipogenesis has significant cell/tissue specificity [87], as it is regulated by a complicated network and works with other core clock products, such as PER1, BMAL1, and REV-ERBα. For example, a substantial reduction in PPARγ is found *in per1/2*−/− mice [88]. However, *Per3* is believed to be a nonessential clock component with modest effects on circadian behaviours [89, 90], and it does inhibit adipogenesis in vivo in a clock manner [91]. The deletion of *Per3* promotes adipogenesis by clock outputs [92]. PER3 binds to PPARγ and inhibits its activity [91, 93]. Increasing PPAR is observed in *Per3*−/− mice, which means that the differentiation arrest resulting from *Per3* happens during the early stages.

D-box binding protein (DBP) and E4 promoter-binding protein 4 (E4BP4), contributors to circadian regulatory loops, interact with the active site of the PPARγ promoter to regulate its rhythmic expression and are enhanced by histone deacetylase inhibitor (HDACi) [94]. Decreased H3K9 acetylation of the DBP promoter leads to a lower level of DBP and a diminished binding between DBP and the PPARγ promoter, which gives rise to a lower level of PPARγ [95].

#### Chondrogenic factors

The circadian clock is critical to early chondrogenesis and endochondral ossification [96, 97]. Indian Hedgehog (*Ihh) is* key in governing chondrogenic differentiation, and its activity is regulated by PER1 and BMAL1 in the growth plate [97]. *Bmal1* deficiency leads to the deregulated rhythmic expression of *Ihh* and *Per* [98]. Our previous studies show that Prg4 and related chondrogenic genes are downstream targets of *Bmal1 in* cartilage formation and chondrogenic differentiation [99]. Cartilage tissue engineering, which combines MSCs with other factors, such as collagen, is a promising therapy for degenerated temporomandibular joint and osteoarthritis. Sry-related HMG box 9 (SOX9), a key transcription regulator of chondrocyte differentiation [100], controls the synthesis of collagen type II, the hallmark of chondrogenic commitment [101]. Impaired circadian rhythm induces osteoarthritis resulting from SOX9 inhibition in articular chondrocytes, and the deletion of *Bmal1* also leads to reduced SOX9 [102] (Table 1). Two additional SOX family members, SOX6 and SOX5, are also involved in chondrogenesis regulation. SOX6 is negatively regulated by its transactivator PER1 (Fig. 2). It shows a long-term decrease after exposure to 10 nM PTH for 1 h in chondrocyte-like ATDC5 cells with no distinct changes in level SOX5 and RUNX2 levels [103].

**Table 1:** Epigenetic modifying enzymes shared both in the circadian clock and in the MSC fate decisions

Circadian component	Epigenetic regulators	Differentiation signals	References
BMAL1-CLOCK	P300/CBP; miR-7-5p	RUNX2	[66]
REV-ERBα/RORα	HDACs	NF-κB	[104]
SIRT1	SIRT1	PPARγ; RUNX2; SOX9; Wnt/β-catenin	[105]
Bmal1	EZH2	BMP2; ATOH8	[106, 107]
Bmal1	DNMT3	Collagen type II	[108]

### The circadian clock determines MSC fate through epigenetic events

Approximately 43% of mammalian genes oscillate in a circadian fashion, but no more than 30% of circadian mRNAs are driven by de novo transcription, indicating that post-transcriptional mechanisms mainly regulate the rhythmic expression of genes in a cell-/tissue-specific fashion [109, 110]. Epigenetics is an essential component of post-transcriptional regulation and has rapidly developed into various fields and disciplines in the twenty-first century, enhancing our understanding of gene regulation in health and disease.

The term ‘epigenetics’ refers to heritable alterations in gene expression without directly altering the nucleotide sequence [111]. The epigenome mediates a crosstalk between cells and the environment through covalent modifications in chromatin (also called epigenetic code) and epigenetic regulators, which include DNA methylation, histone modification, non-coding RNAs, etc. [112]. The epigenome opens or closes chromatin fibre around genes to control subsequent transcription factor binding, exerting great influence on MSC differentiation [113]. The circadian clock has also been implicated in stem cell differentiation, which far exceeds its daily timekeeping roles [114]. Interestingly, accumulating research shows the close relationship between the epigenetic mechanism and circadian rhythm. The rhythmic transcriptome is assisted by a specific epigenetic mechanism which cooperates with the clock to orchestrate daily oscillations [115]. Therefore, we postulate that epigenetic regulators function in the crosstalk between the circadian clock and stem cell fate decisions to determine how and when transcription oscillations occur. The coordinated mechanisms include the clock proteins and epigenetic modifying enzymes constituting complexes to regulate transcription and the collaborative interaction between the clock and non-coding RNAs or chromatin remodellers.

#### Circadian proteins form complexes with epigenetic modifying enzymes

1. **Histone-modifying enzymes**

Genome-wide expression analysis reveals the rhythm of global histone labels that, depending on an intact clock, are under tight regulation by enzymatic feedback loops [109]. The clock recruits epigenetic enzymes to the promoter of important transcription factors and key signalling molecules involved in differentiation. Besides, CLOCK, functioning as an epigenetic modifier, was subsequently found to have a histone acetyltransferase (HAT) activity greatly amplified by its combination with BMAL1 [116]. Additionally, clock-controlled SIRTs and EZH2 provide possibility in elucidating how the clock is linked to epigenetic mechanism in MSCs differentiation.

**Fig. 3:**
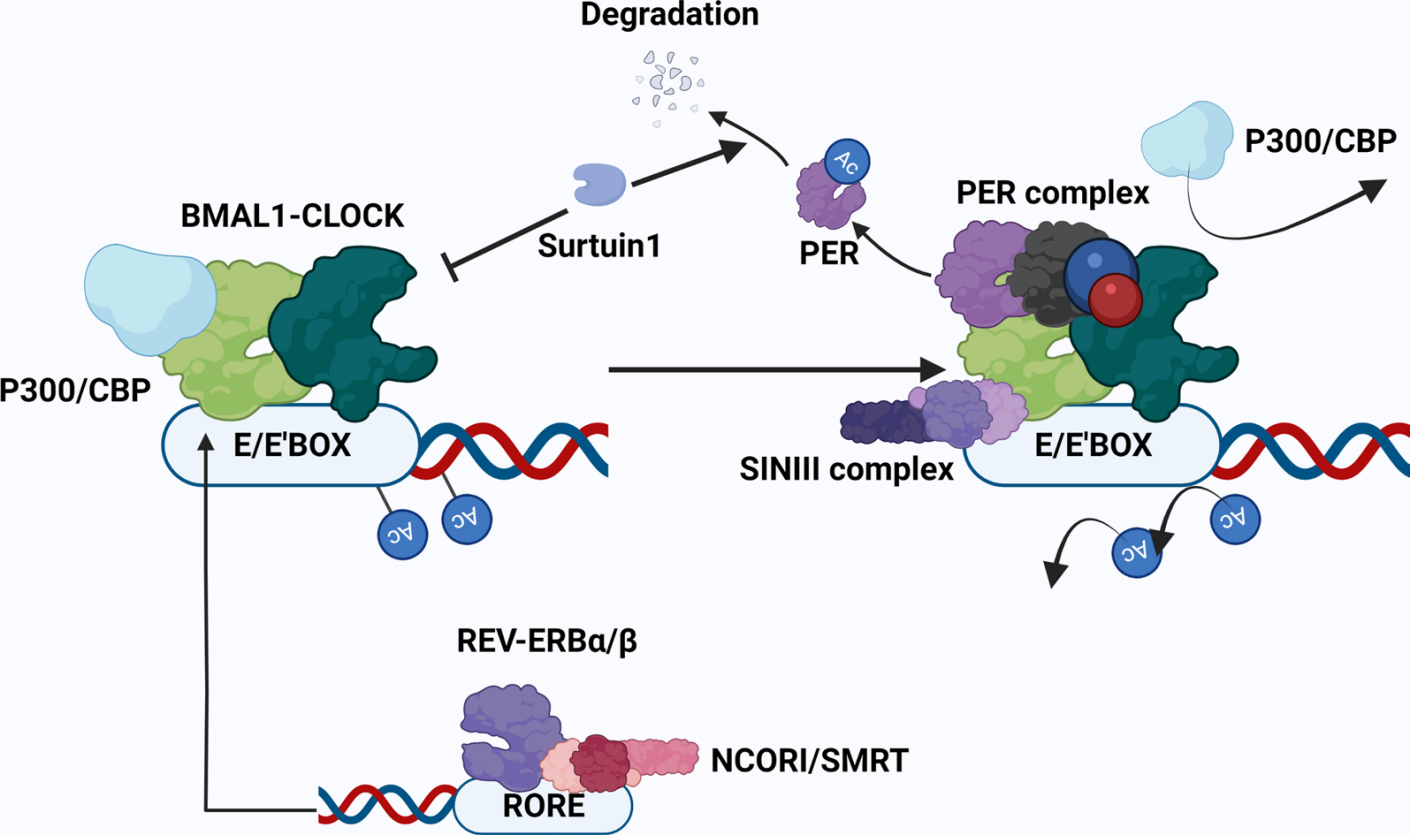
The PER complex, competing with P300/CBP, binds the BMAL1-CLOCK complex. SIRT1 and REV-ERBα/β precisely regulate this process: SIRT1 prompts PER complex dissociation from the BMAL1-CLOCK complex and promotes PER2 degradation. It also plays a negative role in CLOCK HAT activity. REV-ERBα/β recruits the co-repressors NCOR1/SMRT to ROR element to promote P300/CBP binding with the BMAL1-CLOCK complex

At the stage of transcriptional activation, the BMAL1-CLOCK complex acetylates the RUNX2 promoter by Clock itself and by recruiting acetyltransferases P300/CBP, thus promoting osteogenesis in MSCs [66]. At the repression stage, CRY (a constitutive component of the PER complex) occupies the C-terminal transactivation domain of BMAL1, leaving P300/CBP dissociated from its binding site [117]. Once CRY is loaded to the BMAL1-CLOCK complex, it directly recruits the SIN3 complex that comprises HDAC1 and HDAC2 to remove the acetylation tag of promoters (Fig. 3). Thus, the PER-SIN-HDACs pathway targeting histones H3 and H4 controls gene expression precisely and dynamically [118]. In addition, REV-ERBα and RORα exert downstream effects on the expression of NF-kB (which inhibits osteoblast differentiation) by recruiting HDACs [104]. REV-ERBα calls up co-repressors NCOR1/SMRT to ROR element. HDAC3, serving as the catalytic component of NCOR1/SMRT, rhythmically modulates histone deacetylation of the ROR element [119, 120].

Sirtuins (SIRTs) are class III NAD + dependent deacetylases that participate in many biological processes, including the circadian clock and cell differentiation [121–123]. This family has seven members (SIRT1–7) distributed across the cytoplasm, nucleus, and mitochondria. SIRT1 binds BMAL1-CLOCK in a circadian manner and counteracts the HAT activity of CLOCK [124]. Its deacetylation ability extends to BMAL1 and PER2 and relieves BMAL1-CLOCK from CRY and promotes PER2 degradation [125, 126]. SIRT1 requires the cofactors NAD + and NAMPT to function, which are both under tight circadian control [86, 127]. Thus, the deacetylase activity of SIRT1 exhibits a circadian pattern.

The MSC-specific overexpression of SIRT1 increased alveolar bone mass in vivo and promoted MSCs’ proliferation and osteogenic differentiation [128]. Aged mice with a MSC-specific knockout of SIRT1 show reduced subcutaneous fat and a progressive loss of trabecular and cortical bone volume [129]. SIRT1 exerts its effects mainly by deacetylating important transcription factors and signalling pathways, influencing their activity and localisation. For example, SIRT1 directly or indirectly deacetylates RUNX2and PPARγ [130, 131] and deacetylates β-catenin, facilitating its accumulation in the nucleus [132]. Along these lines, SIRT1, serving as a circadian hallmarker, causes dynamic changes in the epigenome to generate transcriptional responses controlling MSC differentiation.

Methylation is also a widespread histone modification in mammalian cells and is linked to transcriptional repression. EZH2, mediating gene silencing via trimethylation of H3K27, is directly regulated by the circadian clock via the E-BOX and RORE motifs in fish species [133]. EZH2 is a repressor of differentiation-associated genes in vitro. It inhibits osteogenesis by inhibiting BMP2 and inhibits chondrogenic differentiation by inhibiting ATOH8 [106, 107] (Table 1). However, in in-vivo animal studies, the additive effects of VDR‐mediated EZH2 transcriptional increase show increased osteogenesis, as EZH2 also inhibits MSC senescence by repressing p16 transcription [134]. MSCs are cells of mesodermal origin which derived from ESCs. The level of EZH2 in MSCs is significantly lower than that in ESCs, and a specific EZH2 inhibitor, GSK126, dramatically drives ESCs differentiation into MSCs by reducing H3K27me3 [135], suggesting that EZH2 levels in specific cell types are crucial for the balance of pluripotency maintenance, cell differentiation, and cell senescence.

(2) **DNA-modifying enzymes**

Tissue-specific DNA methylation patterns play central roles in establishing cell identity during differentiation. Generally, DNA methylation is considered one of the hallmark mechanisms of gene silencing. For example, DNMT inhibitors, such as 5-aza-2'-deoxycytidine, induce gene activation and cell differentiation [136]. However, the genome-wide DNA methylation profiling of MSCs shows an increasing methylation level of the global genome and CpG sites in the process of osteogenic differentiation [137]. These results appear to be incompatible with the gene-silencing role of DNA methylation. Recent studies suggest that the function of DNA methylation varies depending on the methylation location. The methylation of the gene body, and not the promoter, promotes genome stability and even transcription [138], suggesting that promoter segments may not be the DNA methyltransferase target sites during MSCs differentiation.

In recent years, growing evidence in chronobiology hints at a close connection between the circadian rhythm and DNA methylation. DNMTs oscillate in a clock pattern in the mouse liver and brain [139], and excessive exposure to artificial light leads to tissue-specific changes in DNMTs expression [140]. Taken together, this evidence suggests that DNMT may exert great influence during clock-controlled differentiation.

#### Reciprocal regulation between the circadian clock and non-coding RNAs

miRNAs are a class of short non-coding RNAs with a length of about 22 nucleotides. The circadian clock regulates miRNAs expression and activity, and the clock itself is regulated by miRNAs at post-transcriptional levels [141]. And miRNAs taking part in clock regulation have been extensively reviewed [142, 143]. In healthy young males, some circulating miRNAs display 24-h rhythms with two expression patterns: one peaks at night and the other peaks during the daytime [144, 145]. There are 104 miRNAs differently expressed in CLOCK-mutant mice compared with the controlled group, and they are linked to transcription regulation and PI3K-AKT and MAPK signalling pathways [146], which are closely related to MSC differentiation.

However, some researchers argue that mature miRNAs do not oscillate in a circadian pattern. Studies show at least 57 pri-miRNAs oscillated in the mouse liver, with only four mature miRNAs showing the same rhythm as their primary transcript [147]. Interestingly, the rhythmic expression of Dicer (the essential enzyme for miRNA maturation) is found in various tissues [148]. Studies are contradictory and may be model-dependent. The inconsistent findings among these studies can be partly explained by variations in the study samples, methods of assessment, and, most importantly, tissue specificity, which may hint at the role of circadian miRNA in establishing cell identity.

Mounting research has unveiled the importance of miRNAs in the regulation of MSC differentiation. For example, the expression profile in patients with fractures or osteoporosis differs from that in healthy individuals. Hackl et al. summarized the different expression of miRNAs in patients with bone disease and the control group. They rasied the possibilility that circulating miRNAs are used as biomarkers for bone disease [149]. Furthermore, many miRNAs are shared between the circadian clock and MSCs fate decisions. A candidate miRNA set potentially involved in circadian control has been established by various means, including microarrays [150], CHIP [142], and in silico prediction analysis of databases [151]. We found that some of them also function in the differentiation process of MSCs, as summarised in the Additional file 1.

Long non-coding RNAs (lncRNAs) are non-coding RNAs with 2000–100,000 nucleotides in length. A mouse transcriptome analysis indicated that more than 1000 lncRNAs show a diurnal rhythm [152]. The expression of a substantial portion of rhythmic lncRNAs is pronounced in regions of enhancer clusters and regulates gene expression, showing high similarity in rhythm phase and expression patterns with genes in their proximity. For instance, lnc-CROT is highly expressed at super-enhancer regions and modulates long-range circadian gene regulation [153]. Accumulating research has shown that lncRNAs, such as lnc-MEG3 and lnc-XIST, take part in osteogenic differentiation [154, 155]. Unfortunately, there are few reports on the role of lncRNAs in the clock-controlled differentiation process, and follow-up bioinformatics studies should be carried out.

#### Circadian proteins influencing chromatin architecture

Different kinds of epigenetic mechanisms show a pile-up effect on gene expression levels by influencing chromatin architecture and accessibility, which influence the binding of transcription factors to the genome. The molecular clock, working with various epigenetic regulators, contributes to spatiotemporal balance and the oscillation of chromatin accessibility [109, 156]. For example, RORα/β, BMAL1, and other circadian proteins promote global chromatin decondensation during the activation phase via the SWI/SNF complex (one of the epigenetic remodelling complexes). In turn, this promotes the load of REV-ERB, which rapidly responds to structure changes and inhibits circadian protein expression [157]. Furthermore, the chromatin landscape shaped by circadian regulation is critical for MSCs self-renewal and multilineage differentiation [158].

## Future perspective

Circadian dysfunction is associated with various diseases in mammals [159], such as myocardial infarctions (MIs) and bone diseases [160]. Interrupted light at night causes depressive-like behaviours in mice by the circadian ipRGCs-dpHb-NAc (retinal ganglion cells–the dorsal perihabenular nucleus–the nucleus accumbens) pathway [161]. Besides, the symptom complexity and the onset of various diseases show marked circadian rhythm. For example, the incidence of MIs at 9 a.m. is three times greater than that at 11 p.m., periods associated with the blood pressure rhythm [162]. Thus, manipulating and maintaining the circadian rhythm is important for disease prevention. The circadian rhythm of disease onset and chrono-drug discovery should be taken into account when deciding treatment times [163]**.**

Luckily, the circadian rhythm can be remodelled by environmental cues. Light, temperature, eating frequency, and regular exercises are proved to be daily circadian temporal cues [164, 165]. Regular exercise rescued the disrupted circadian rhythm by increasing *Per2* expression amplitude [166]. Time-restricted feeding increasing circadian amplitude prevents metabolic diseases and obesity in mice fed a high-fat diet [167]. Short-term caloric restriction is associated with cardio-protection [168]. This suggests that we may alter MSC function and restore bone function and structure by rectifying eating frequency and the sleep–wake pattern and reducing night-time exposure to artificial light to reset the circadian clock.

In addition to behavioural improvements, mechanical stress, biotic factors, and small molecule modifiers are promising interventions for a robust rhythm. Mechanical stress regulates the clock of skeletal muscle cells (C2C12 cells) by decreasing Per/Cry expression and increasing Bmal1 expression [169]. The Per1 inhibition of BMSC by artificial materials can be rescued by vitamin D supplements and osteogenic media [170]. Melatonin supplements, overcoming rhythm disruption, exhibit excellent treatment efficacy and safety as ideal candidates for preventing bone loss. GCs like dexamethasone are widely used to generate synchronised clocks in vitro. Some small molecule modifiers of circadian clocks alter the circadian period length, phase, and amplitude with favourable benefits. For example, *Rev-erb* activation by SR9009 is beneficial for the reparative process after ischaemia–reperfusion injury by attenuating NLRP3 inflammasome activation and immune infiltration, being also essential for protection against the development of cardiac hypertrophy and heart failure in mice [171, 172]. Furthermore, the proliferation and differentiation of neural stem cells are promoted by SR9009 in a dose-dependent manner through targeting *Rev-erb* [173]. However, clock is found in almost all tissues in mammals, and systematic influences on clock activity may interrupt normal physiological processes in healthy tissues. Thus, the specificity and safety of those modifiers should be fully reviewed before it is widely applied in clinical practice.

Additionally, different stem cells are sensitive to specific entraining cues with different circadian patterns. In DPSCs, mechanosensitive synchronisation is more accessible than dexamethasone synchronisation. BMSCs are more responsive to chemical stimulation than dexamethasone, and stem cells show different responses to entraining cues at certain times of the day [10, 162]. This hints at specific entraining choices for the circadian rhythm of different cells at specific times.

Our study has several limitations, since the interplay between the circadian rhythm and cell differentiation is a complicated network rather than a unidirectional hierarchical structure. How chromatin modifiers [174], systemic conditions, signalling pathways, kinases and phosphatases [175], RNA-binding factors [176], and other factors are involved in the whole regulatory network remains to be identified. However, the development of high-throughput techniques and novel sequencing technologies makes it possible to depict this underlying network, which is beneficial for a deeper understanding of MSC [1]. We may potentially improve MSC differentiation efficiency for future clinical applications.

## Conclusions

The connections between the circadian rhythm and cell differentiation have been intensively researched in recent years. The underlying associated mechanisms remain to be summarised. This review elaborates on key coupling mechanisms, which include the following: (1) The circadian clock affects hormone secretion to regulate cell differentiation; (2) molecular clock core products directly modulate the activity and expression of key transcription factors; and (3) histone modifications, DNA modification, non-coding RNAs, and large multi-subunit chromatin remodelling complexes and other epigenetic modifications are critical nodes in the circadian regulation of cell fate decision. We offer an in-depth insight into the interplay between the circadian clock and cell differentiation in the skeletal system. Additionally, we suggest that incorporating circadian regulation (ranging from chemical, biological, and physical cues) may have profound benefits in optimising tissue engineering approaches and stem cell therapies.

## Supplementary Information

**Additional file 1**. Predicted clock-targeted miRNAs in mice and human which also working in the MSC differentiation

## Data Availability

All data generated or analysed during this study are included in this published article.

## Abbreviations

SCN: Suprachiasmatic nuclei
Bmal1: Brain and muscle ARNT-like protein 1
Per: Period
Cry: Cryptochrome
RORα/β/γ: Retinoid-related orphan receptor α/β/γ
MSCs: Mesenchymal stem cells
ESCs: Embryonic stem cells
iPSCs: Induced pluripotent stem cells
BMSCs: Bone marrow stem cells
ADSCs: Adipose tissue-derived mesenchymal stem cells
DPSCs: Dental pulp stem cells
PTH: Parathyroid hormone
BMAT: Bone marrow adipose tissue
GCs: Glucocorticoids
SOX9: Sry-related HMG box 9
PPAR γ: Peroxisome proliferator-activated receptor γ
TZD: Thiazolidinedione
CCGs: Clock-controlled genes
ATF4: CAMP-dependent transcription factor 4
FOXO: Forkhead box O
NADP + /NADPH: Nicotinamide adenine dinucleotide phosphate
DBP: D-box binding protein
E4BP4: E4 promoter-binding protein 4
CBP: CREB-binding protein
HDACs: Histone deacetylases
SIRTs: Sirtuins
EZH2: Histone-lysine N-methyltransferase
DNMTs: DNA methyltransferases
CpG: Cytosine-phosphate-guanine
lncRNAs: Long non-coding RNAs
MIs: Myocardial infarctions
ipRGCs-dpHb-NAc: Retinal ganglion cells–the dorsal perihabenular nucleus–the nucleus accumbens
