# Supplementary material for: The circadian clock has roles in mesenchymal stem cell fate decision
Source: Stem Cell Res Ther. 2022 May 16;13:200. doi: 10.1186/s13287-022-02878-0 (PMC9109355; doi:10.1186/s13287-022-02878-0)
Supplement: Supplementary file 1 — Additional file 1. Predicted clock-targeted miRNAs in mice and human which also working in the MSC differentiation [file 13287_2022_2878_MOESM1_ESM.docx]

|  |
| --- |

|  |
| --- |

|  |
| --- |
|  |

Additional file 1. Predicted clock-targeted miRNAs in mice and human which also working in the MSC differentiation.

| miRNA | Predicted Targets for mouse | Predicted Targets for Human | Prediction source of miRNA-clock | The role of miRNA in differenation | References |  |  |  |  |  |  |
| --- | --- | --- | --- | --- | --- | --- | --- | --- | --- | --- | --- |
| Let-7a | *Cry2;Rorc;Ror-γ* | Cry2 | ^1^ | (-)osteogenesis | ^2^ |  |  |  |  |  |  |
| Let-7b | *Cry2;Rorc;Ror-γ* | Cry2 | ^1^ | (-)osteogenesis | ^3, 4^ |  |  |  |  |  |  |
| Let-7c | *Cry2;Rorc;Ror-γ* | Cry2 | ^1^ | (±)osteogenesis | ^5, 6^ |  |  |  |  |  |  |
| Let-7f | *Cry2;Rorc;Ror-γ* | Cry2 | ^1^ | (+)osteogenesis | ^7^ {Shen, 2018 #1480} |  |  |  |  |  |  |
| miR-106a | *Clock;Cry2;Ccnd1* | Cry2 | ^1^ | (-) osteogenesis | ^8^ |  |  |  |  |  |  |
| miR-106b | *Clock;Cry2;Ccnd1* | Cry2 | ^1^ | (-)osteogenesis | ^9^ |  |  |  |  |  |  |
| miR-146b | *Cry1;Per3;Timeless* | \ | ^10^ | (-)chondrogenic differentiation | ^11^ |  |  |  |  |  |  |
| miR-152 | *Clock;Grp58* | Clock;Grp58 | ^1^ | (-)osteogenesis | ^12^ |  |  |  |  |  |  |
| miR-16 | *Ccne1* | Clock | ^1^ | (-)osteogenesis | ^13^ |  |  |  |  |  |  |
| miR-181a | *Clock;Timeless* | \ | ^10^ | (-)osteogenesis | ^14^ |  |  |  |  |  |  |
| miR-181d | *Clock;Timeless* | \ | ^10^ | (-)osteogenesis | ^15^ |  |  |  |  |  |  |
| miR-182 | *Clock;Adcy6* | Clock;Adcy6 | ^1^ | (-)osteogenesis  (-)chondrogenic differentiation | ^16, 17^ |  |  |  |  |  |  |
| miR-195 | *Ccne1* | Clock;Ccnd1;Ccne1 | ^1^ | (-)osteogenesis | ^18^ |  |  |  |  |  |  |
| miR-203 | *Bmal1* | Bmal1 | ^1^ | (±)osteogenesis;  (-)adipogenesis | ^19, 20^ |  |  |  |  |  |  |
| miR-204 | *Adcy6* | Adcy6 | ^1^ | (-)osteogenesis | ^21^ |  |  |  |  |  |  |
| miR-211 | *Adcy6* | Adcy6 | ^1^ | (+)osteogenesis  (+)chondrogenic differentiation | ^22, 23^ |  |  |  |  |  |  |
| miR-212 | *Hao1* | Hao1 | ^1^ | (-)osteogenesis | ^24^ |  |  |  |  |  |  |
| miR-24 | *Per2* | Per1;Per2 | ^1^ | (-)osteogenesis | ^25^ |  |  |  |  |  |  |
| miR-25 | *Per2* | Per2 | ^1^ | (+)osteogenesis | ^26^ |  |  |  |  |  |  |
| miR-26a | *Hao1* | Hao1 | ^1^ | (±)osteogenesis | ^27^ |  |  |  |  |  |  |
| miR-27a | *Adcy6* | Adcy6 | ^1^ | (+)osteogenesis | ^28^ |  |  |  |  |  |  |
| miR-27b | *Adcy6* | Adcy6 | ^1^ | (+)osteogenesis | ^29^ |  |  |  |  |  |  |
| miR-29a | *Per3* | Per3 | ^1^ | (+)osteogenesis;(-)adipogenesis | ^30^ |  |  |  |  |  |  |
| miR-29b | *Per3* | Per3 | ^1^ | (-)osteogenesis; | ^31^ |  |  |  |  |  |  |
| miR-302a | *Ror-β;Ccnd1* | Ror-β;Ccnd1 | ^1^ | (+)osteogenesis | ^32^ |  |  |  |  |  |  |
| miR-30b | *Per2* | Per2 | ^1^ | (-)osteogenesis | ^33^ |  |  |  |  |  |  |
| miR-30d | *Clock;Per2* | Clock;Per2 | ^1^ | (-)osteogenesis | ^34^ |  |  |  |  |  |  |
| miR-30e | *Clock;Per2* | Clock;Per2 | ^1^ | (-)osteogenesis  (+) adipogenesis | ^35^ |  |  |  |  |  |  |
| miR-324-3p | *Npas2;Rev-erbα* | \ | ^10^ | (+)osteogenesis | ^36^ |  |  |  |  |  |  |
| miR-346 | *Csnk1D* | Csnk1D | ^1^ | (+)osteogenesis | ^37^ |  |  |  |  |  |  |
| miR-365 | Clock | \ | ^10^ | (+)chondrogenic differentiation | ^38^ |  |  |  |  |  |  |
| miR-383 | *Cry1* | \ | ^10^ | (-)osteogenesis | ^39^ |  |  |  |  |  |  |
| miR-483 | *Cry1* | \ | ^10^ | (+)osteogenesis | ^40^ |  |  |  |  |  |  |
| miR-494 | *Bmal1* | \ | ^41^ | (-)osteogenesis | ^42^ |  |  |  |  |  |  |
| miR-93 | *Clock;Cry2;Ccnd1* | Clock;Cry2;Ccnd1 | ^1^ | (-)osteogenesis | ^43^ |  |  |  |  |  |  |
| miR-98 | *Cry2;Rorc* | Cry2 | ^1^ | (-)osteogenesis | ^44^ |  |  |  |  |  |  |

|  |
| --- |

References:

1. Figueredo DdS, Barbosa MR, Gitaí DLG, et al. Predicted microRNAs for mammalian circadian rhythms. Journal of Biological Rhythms. 2013;28(2):107-116.

2. Ma W, Dou Q, Ha X. Let-7a-5p inhibits BMSCs osteogenesis in postmenopausal osteoporosis mice. Biochemical and Biophysical Research Communications. 2019;510(1):53-58.

3. Fu L, Li N, Ye Y, et al. MicroRNA Regulates the Osteogenic Differentiation of Human Periodontal Ligament Stem Cells by Targeting CTHRC1. Stem Cells International. 2021;2021:5791181.

4. Wang L-J, Cai H-Q. Let-7b downgrades CCND1 to repress osteogenic proliferation and differentiation of MC3T3-E1 cells: An implication in osteoporosis. The Kaohsiung Journal of Medical Sciences. 2020;36(10):775-785.

5. Liu G-X, Ma S, Li Y, et al. Hsa-let-7c controls the committed differentiation of IGF-1-treated mesenchymal stem cells derived from dental pulps by targeting IGF-1R via the MAPK pathways. Experimental & Molecular Medicine. 2018;50(4).

6. Yuan H, Zhao H, Wang J, et al. MicroRNA let-7c-5p promotes osteogenic differentiation of dental pulp stem cells by inhibiting lipopolysaccharide-induced inflammation via HMGA2/PI3K/Akt signal blockade. Clinical and Experimental Pharmacology & Physiology. 2019;46(4):389-397.

7. Shen G-Y, Ren H, Shang Q, et al. Let-7f-5p regulates TGFBR1 in glucocorticoid-inhibited osteoblast differentiation and ameliorates glucocorticoid-induced bone loss. International Journal of Biological Sciences. 2019;15(10):2182-2197.

8. Jia B, Qiu X, Chen J, et al. A feed-forward regulatory network lncPCAT1/miR-106a-5p/E2F5 regulates the osteogenic differentiation of periodontal ligament stem cells. Journal of Cellular Physiology. 2019;234(11):19523-19538.

9. Fang T, Wu Q, Zhou L, et al. miR-106b-5p and miR-17-5p suppress osteogenic differentiation by targeting Smad5 and inhibit bone formation. Experimental Cell Research. 2016;347(1):74-82.

10. Na YJ, Sung JH, Lee SC, et al. Comprehensive analysis of microRNA-mRNA co-expression in circadian rhythm. Experimental & Molecular Medicine. 2009;41(9):638-647.

11. Budd E, de Andrés MC, Sanchez-Elsner T, et al. MiR-146b is down-regulated during the chondrogenic differentiation of human bone marrow derived skeletal stem cells and up-regulated in osteoarthritis. Scientific Reports. 2017;7:46704.

12. Feng L, Xia B, Tian B-F, et al. MiR-152 influences osteoporosis through regulation of osteoblast differentiation by targeting RICTOR. Pharmaceutical Biology. 2019;57(1):586-594.

13. Duan L, Zhao H, Xiong Y, et al. miR-16-2 Interferes with WNT5A to Regulate Osteogenesis of Mesenchymal Stem Cells. Cell Physiol Biochem. 2018;51(3):1087-1102.

14. Zhu H, Chen H, Ding D, et al. The interaction of miR-181a-5p and sirtuin 1 regulated human bone marrow mesenchymal stem cells differentiation and apoptosis. Bioengineered. 2021;12(1):1426-1435.

15. Xie Y, Hu JZ, Shi ZY. MiR-181d promotes steroid-induced osteonecrosis of the femoral head by targeting SMAD3 to inhibit osteogenic differentiation of hBMSCs. Eur Rev Med Pharmacol Sci. 2018;22(13):4053-4062.

16. Bai M, Yin H, Zhao J, et al. miR-182-5p overexpression inhibits chondrogenesis by down-regulating PTHLH. Cell Biology International. 2019;43(3):222-232.

17. Chen D, Xiang M, Gong Y, et al. LIPUS promotes FOXO1 accumulation by downregulating miR-182 to enhance osteogenic differentiation in hPDLCs. Biochimie. 2019;165:219-228.

18. Chao C, Li F, Tan Z, et al. miR-195 inhibited abnormal activation of osteoblast differentiation in MC3T3-E1 cells via targeting RAF-1. Experimental Cell Research. 2018;362(2):293-301.

19. Xia ZL, Wang Y, Sun QD, et al. MiR-203 is involved in osteoporosis by regulating DKK1 and inhibiting osteogenic differentiation of MSCs. Eur Rev Med Pharmacol Sci. 2018;22(16):5098-5105.

20. Qiao L, Liu D, Li CG, et al. MiR-203 is essential for the shift from osteogenic differentiation to adipogenic differentiation of mesenchymal stem cells in postmenopausal osteoporosis. Eur Rev Med Pharmacol Sci. 2018;22(18):5804-5814.

21. Zhou Y, Liu S, Wang W, et al. The miR-204-5p/FOXC1/GDF7 axis regulates the osteogenic differentiation of human adipose-derived stem cells via the AKT and p38 signalling pathways. Stem Cell Research & Therapy. 2021;12(1):64.

22. Liu H, Luo J. miR-211-5p contributes to chondrocyte differentiation by suppressing Fibulin-4 expression to play a role in osteoarthritis. J Biochem. 2019;166(6):495-502.

23. Gong Z-M, Tang Z-Y, Sun X-L. LncRNA PRNCR1 regulates osteogenic differentiation in osteolysis after hip replacement by targeting miR-211-5p. Biosci Rep. 2018.

24. Zhang Y, Jiang Y, Luo Y, et al. Interference of miR-212 and miR-384 promotes osteogenic differentiation via targeting RUNX2 in osteoporosis. Exp Mol Pathol. 2020;113:104366.

25. Li Z, Sun Y, Cao S, et al. Downregulation of miR-24-3p promotes osteogenic differentiation of human periodontal ligament stem cells by targeting SMAD family member 5. Journal of Cellular Physiology. 2019;234(5):7411-7419.

26. Li X, Ji J, Wei W, et al. MiR-25 promotes proliferation, differentiation and migration of osteoblasts by up-regulating Rac1 expression. Biomedicine & Pharmacotherapy = Biomedecine & Pharmacotherapie. 2018;99:622-628.

27. Su X, Liao L, Shuai Y, et al. MiR-26a functions oppositely in osteogenic differentiation of BMSCs and ADSCs depending on distinct activation and roles of Wnt and BMP signaling pathway. Cell Death & Disease. 2015;6:e1851.

28. Guo D, Li Q, Lv Q, et al. MiR-27a targets sFRP1 in hFOB cells to regulate proliferation, apoptosis and differentiation. PloS One. 2014;9(3):e91354.

29. Seenprachawong K, Tawornsawutruk T, Nantasenamat C, et al. miR-130a and miR-27b Enhance Osteogenesis in Human Bone Marrow Mesenchymal Stem Cells via Specific Down-Regulation of Peroxisome Proliferator-Activated Receptor γ. Front Genet. 2018;9:543.

30. Ko J-Y, Chuang P-C, Ke H-J, et al. MicroRNA-29a mitigates glucocorticoid induction of bone loss and fatty marrow by rescuing Runx2 acetylation. Bone. 2015;81:80-88.

31. Zhang Y, Chen B, Li D, et al. LncRNA NEAT1/miR-29b-3p/BMP1 axis promotes osteogenic differentiation in human bone marrow-derived mesenchymal stem cells. Pathol Res Pract. 2019;215(3):525-531.

32. Kang I-H, Jeong B-C, Hur S-W, et al. MicroRNA-302a stimulates osteoblastic differentiation by repressing COUP-TFII expression. Journal of Cellular Physiology. 2015;230(4):911-921.

33. Liu G, Lu Y, Mai Z, et al. Suppressing MicroRNA-30b by Estrogen Promotes Osteogenesis in Bone Marrow Mesenchymal Stem Cells. Stem Cells International. 2019;2019:7547506.

34. Wu Z-H, Huang K-H, Liu K, et al. DGCR5 induces osteogenic differentiation by up-regulating Runx2 through miR-30d-5p. Biochemical and Biophysical Research Communications. 2018;505(2):426-431.

35. Ding W, Li J, Singh J, et al. miR-30e targets IGF2-regulated osteogenesis in bone marrow-derived mesenchymal stem cells, aortic smooth muscle cells, and ApoE-/- mice. Cardiovascular Research. 2015;106(1):131-142.

36. Razny U, Polus A, Goralska J, et al. Effect of insulin resistance on whole blood mRNA and microRNA expression affecting bone turnover. European Journal of Endocrinology. 2019;181(5):525-537.

37. Wang Q, Cai J, Cai X-H, et al. miR-346 regulates osteogenic differentiation of human bone marrow-derived mesenchymal stem cells by targeting the Wnt/β-catenin pathway. PloS One. 2013;8(9):e72266.

38. Chen J, Wu X. Cyclic tensile strain promotes chondrogenesis of bone marrow-derived mesenchymal stem cells by increasing miR-365 expression. Life Sciences. 2019;232:116625.

39. Tang J, Zhang Z, Jin X, et al. miR-383 negatively regulates osteoblastic differentiation of bone marrow mesenchymal stem cells in rats by targeting Satb2. Bone. 2018;114:137-143.

40. Xiao Y, Guo Q, Jiang T-J, et al. miR‑483‑3p regulates osteogenic differentiation of bone marrow mesenchymal stem cells by targeting STAT1. Molecular Medicine Reports. 2019;20(5):4558-4566.

41. Mehta N, Cheng H-YM. Micro-managing the circadian clock: The role of microRNAs in biological timekeeping. Journal of Molecular Biology. 2013;425(19):3609-3624.

42. Qin W, Liu L, Wang Y, et al. Mir-494 inhibits osteoblast differentiation by regulating BMP signaling in simulated microgravity. Endocrine. 2019;65(2):426-439.

43. Zhang Y, Zhuang Z, Wei Q, et al. Inhibition of miR-93-5p promotes osteogenic differentiation in a rabbit model of trauma-induced osteonecrosis of the femoral head. FEBS Open Bio. 2021.

44. Zhang G-P, Zhang J, Zhu C-H, et al. MicroRNA-98 regulates osteogenic differentiation of human bone mesenchymal stromal cells by targeting BMP2. Journal of Cellular and Molecular Medicine. 2017;21(2):254-264.
